# 2226. Analysis of Co-Resistance Among *Klebsiella pneumoniae* Urine Isolates From Female Outpatients in the United States

**DOI:** 10.1093/ofid/ofac492.1845

**Published:** 2022-12-15

**Authors:** Keith S Kaye, Vikas Gupta, Aruni Mulgirigama, Ashish V Joshi, Nicole E Scangarella-Oman, Kalvin Yu, Janet Watts, Fanny S Mitrani-Gold

**Affiliations:** Rutgers - Robert Wood Johnson Medical School, New Brunswick, New Jersey; Becton, Dickinson and Company (BD), Franklin Lakes, New Jersey; GlaxoSmithKline plc., Surrey, England, United Kingdom; GlaxoSmithKline plc., Surrey, England, United Kingdom; GlaxoSmithKline plc., Surrey, England, United Kingdom; Becton, Dickinson and Company (BD), Franklin Lakes, New Jersey; Becton, Dickinson and Company (BD), Franklin Lakes, New Jersey; GlaxoSmithKline plc., Surrey, England, United Kingdom

## Abstract

**Background:**

Over the past decade, extended spectrum β-lactamase producing Enterobacterales (ESBL) and multidrug resistance has risen among community-acquired uncomplicated urinary tract infections (uUTIs). This study was conducted to determine co-resistance among *Klebsiella pneumoniae* (*K. pneumoniae*) isolates in urine from female outpatients in the United States (US).

**Methods:**

This was a retrospective, cross-sectional study of 30-day non-duplicate *K. pneumoniae* urine isolates from female outpatients (≥ 12 years of age) at 304 US facilities, with ≥ 3 months of data from 2011–2019 (Becton, Dickinson and Company [BD] Insights Research Database). *K. pneumoniae* isolates were defined as those that were ESBL-positive (ESBL+) (by commercial panel or not susceptible [NS] to ceftriaxone, cefotaxime, ceftazidime or cefepime), or NS if intermediate/resistant to any of the following: fluoroquinolones (FQs), trimethoprim/sulfamethoxazole (SXT), nitrofurantoin (NFT). Co-resistance phenotypes were characterized in isolates NS to ≥ 2 of the 4 resistance phenotypes assessed.

**Results:**

Among 250,719 non-duplicate *K. pneumoniae* isolates evaluated, 11,065 were ESBL+ (Table). Co-resistance among ESBL+ isolates was observed as follows: 54.9% to FQ; 65.7% to SXT; 75.5% to NFT; and 38.2% to all 4 resistance phenotypes. Of 10,962 isolates NS to FQ, co-resistance was observed as follows: 55.4% were ESBL+; 65.7% to SXT; 79.6% to NFT; and 38.6% to all 4 resistance phenotypes. Of 141,545 isolates NS to NFT, co-resistance was observed as follows: 5.9% were ESBL+; 6.2% to FQ; 11.8% to SXT; and 3.0% to all 4 resistance phenotypes. Among 23,887 isolates NS to SXT, co-resistance was observed as follows: 30.4% were ESBL+; 30.1% to FQ; 69.7% to NFT; and 17.7.% to all 4 resistance phenotypes.

**Table.**
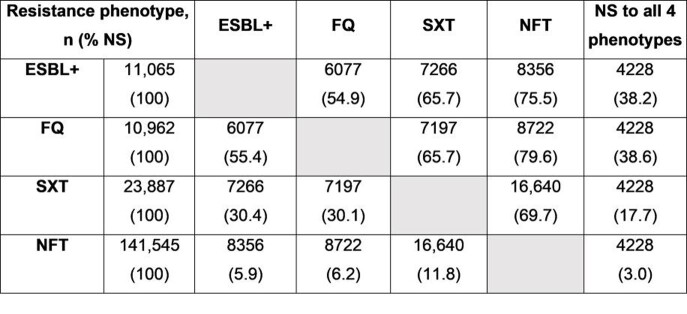
Co-resistance phenotype combinations observed among Klebsiella pneumoniae isolates in urine from 2011–2019

**Conclusion:**

The high prevalence of co-resistance in 30-day non-duplicate outpatient *K. pneumoniae* urinary isolates, particularly towards NFT (70–80% of isolates), limits the effective oral treatment options for uUTI. These analyses may help inform and optimize appropriate empiric treatment of female outpatients with uUTI in the US.

**Disclosures:**

**Keith S. Kaye, MD, MPH**, Allecra: Advisor/Consultant|GlaxoSmithKline plc.: Receiving symposia honoraria|GlaxoSmithKline plc.: GlaxoSmithKline plc.-sponsored study 212502|Merck: Advisor/Consultant|qpex: Advisor/Consultant|Shionogi: Grant/Research Support|Spero: Advisor/Consultant **Vikas Gupta, PharmD**, Becton, Dickinson and Company: Employee of, and shareholder in, Becton, Dickinson and Company, and the company received funding from GlaxoSmithKline plc. to conduct this study|GlaxoSmithKline plc.: GlaxoSmithKline plc.-sponsored study 212502 **Aruni Mulgirigama, MBBS**, GlaxoSmithKline plc.: Employee and shareholder|GlaxoSmithKline plc.: GlaxoSmithKline plc.-sponsored study 212502 **Ashish V. Joshi, PhD**, GlaxoSmithKline plc.: Employee and shareholder|GlaxoSmithKline plc.: GlaxoSmithKline plc.-sponsored study **Nicole E. Scangarella-Oman, MS**, GlaxoSmithKline plc.: Employee and shareholder **Kalvin Yu, MD, FIDSA**, Becton, Dickinson and Company: Employee of, and shareholder in, Becton, Dickinson and Company, and the company received funding from GlaxoSmithKline plc. to conduct this study|GlaxoSmithKline plc.: GlaxoSmithKline plc.-sponsored study 212502 **Janet Watts, PhD**, Becton, Dickinson and Company: Employee of Becton, Dickinson and Company, and the company received funding from GlaxoSmithKline plc. to conduct this study|GlaxoSmithKline plc.: GlaxoSmithKline plc.-sponsored study 212502 **Fanny S. Mitrani-Gold, MPH**, GlaxoSmithKline plc.: Employee and shareholder|GlaxoSmithKline plc.: GlaxoSmithKline plc.-sponsored study 212502.

**Table.** Co-resistance phenotype combinations observed among Klebsiella pneumoniae isolates in urine from 2011–2019

In total, 250,719 non-duplicate (30-day) Klebsiella pneumoniae isolates were evaluated.

Note: Some isolates had overlapping susceptibilities/antimicrobial resistance types, hence individual phenotype totals do not reflect total number of isolates evaluated.

Abbreviations: ESBL+, extended spectrum β-lactamase-producing or not susceptible to ceftriaxone, cefotaxime, ceftazidime or cefepime; FQ, fluoroquinolone; NFT, nitrofurantoin; NS, not susceptible; SXT, trimethoprim/sulfamethoxazole.

